# Association between Stress and Physical Fitness of University Students Post-COVID-19 Pandemic

**DOI:** 10.3390/jfmk8010033

**Published:** 2023-03-02

**Authors:** Boonsita Suwannakul, Noppharath Sangkarit, Pacharee Manoy, Patchareeya Amput, Weerasak Tapanya

**Affiliations:** 1 Department of Physical Therapy, School of Allied Health Science, University of Phayao, Phayao 56000, Thailand; 2 Unit of Excellence of Human Performance and Rehabilitations, University of Phayao, Phayao 56000, Thailand

**Keywords:** stress, physical fitness, university students, post-COVID-19 pandemic

## Abstract

Post-COVID-19 pandemic, most universities changed their educational model from online courses to onsite learning, allowing students to attend regular face-to-face classes. These changes can cause stress in students, which affects physical fitness. The aim of this study was to investigate the relationship between stress levels and physical fitness in female university students. The participants were 101 female university students, 18–23 years of age. All participants completed the Suan Prung Stress Test-60 (SPST-60). The physical fitness test included body composition, cardiorespiratory fitness, as well as musculoskeletal fitness. Multiple linear regression analysis was used to determine the associations between SPST-60 scores and physical fitness. A *p*-value < 0.05 was considered statistically significant. We found a negative correlation between the sources of stress scores, here environment, and maximal oxygen consumption (β = −0.291; 95% CI, −0.551, −0.031). We also found that symptoms of stress scores in the parasympathetic and sympathetic nervous systems were positively associated with waist-hip circumference ratio (WHR) (β = 0.010; 95% CI, 0.002, 0.017 and β = 0.006; 95% CI, 0.000, 0.012, respectively). Moreover, the symptoms of stress, here emotion, were positively associated with the WHR (β = 0.005; 95 %CI, 0.001, 0.009) and negatively associated with upper extremity muscle strength (β = −0.005; 95% CI, −0.009, 0.000). The results of this study confirmed the associations between stress levels in the post-COVID-19 pandemic era and WHR, maximal oxygen consumption, and upper extremity muscle strength. As a result, stress reduction or prevention alternatives should be considered in order to maintain physical fitness and prevent stress disorders.

## 1. Introduction

The novel coronavirus disease 2019 (COVID-19) triggered a global pandemic in 2020. Most universities canceled face-to-face classes and closed campuses to prevent the spread of the virus. In the post-COVID-19 era, by 2021–2022, most universities switched from online classes to a hybrid model or onsite learning, allowing students to attend residential classes as usual. People are still afraid of becoming infected because the pandemic is not over. While COVID-19 remains widespread, these changes trigger coping behaviors in response to stressful situations. People still follow the public authorities’ preventive guidelines, such as social distancing, wearing a facemask, washing their hands, and an ongoing new normal living style [1,2]. The fourth wave of the COVID-19 pandemic caused by the Omicron strain began in Thailand on 1 January 2022 and has lasted to the present. The mortality rate of patients infected with the Omicron strain, on the other hand, was four times lower than that of the Delta strain, which was widely spread in the pandemic’s third wave. Furthermore, the rate of receiving the COVID-19 vaccine is quite high (data on 28 March 2022; first shot: 79.5%, second shot: 72.2%, and third shot: 33.3%). The Thai government decided to plan for an endemic approach to COVID-19 to provide normal living and encourage social and economic development [3]. They declared that Thailand would be post-pandemic after 30 June 2022. Therefore, most universities opened their doors again. University students have been highly vulnerable to stress and other mental health issues from these changes. A study on the mental health of college students in the post-COVID-19 era found that the risk of post-traumatic stress disorder (PTSD) was increased by living in a rural area, the fear of taking public transport, and the decline of family relationships [4]. The prevalence of fatigue was high among Chinese nursing students in the post-COVID-19 era [5]. In addition, financial distress and infection were significantly associated with depression and anxiety among US college students [6].

Stress has a pernicious effect on both physical and psychological health outcomes. Stress causes mental symptoms such as cognitive dysfunction, dementia, and excessive fatigue [7,8]. There is evidence that stress decreases physical activity [9,10,11]. The experience of stress impairs efforts to be physically active [12]. Both stress and a sedentary lifestyle could have adverse impacts on physical fitness. Chronic stress increases vasoconstriction resulting in high blood pressure and hypertrophy of the left ventricle. These symptoms cause cardiac arrhythmias and subsequent myocardial infarction [13,14,15]. In addition, stress hormones cause catabolism of muscle protein and induce oxidative damage, thereby decreasing muscle strength, quality, and function [16,17]. Chronic stress influences dietary intake, such as the consumption of energy-dense food, which could increase fat mass [18]. In addition, previous studies reported that stress correlated with obesity, which contributes to cardiovascular disease [19,20]. 

The purpose of this study was to examine any associations between stress levels in female university students, assessed using the Suan Prung Stress Test-60 (SPST-60), and physical fitness, which included body composition, cardiorespiratory fitness, and muscular strength and flexibility. We hypothesized that the changes in the educational model and lifestyle of the university students post-COVID-19 pandemic might induce stress levels and be associated with their physical fitness. 

## 2. Materials and Methods

### 2.1. Study Population

This cross-sectional survey study was conducted during the first term of 2022 (from July to August 2022). The participants were female university students at the Department of Physical Therapy, School of Allied health Science, University of Phayao, Northern Thailand, who enrolled for the first trimester of the academic year 2022–2023. Eligibility criteria included being 18–22 years old, healthy, and having no major adverse health conditions that would affect cardiorespiratory and neuromuscular fitness (such as heart disease, asthma, hypertension, and myopathy). A total of 193 female physical therapy students were recruited. There were 105 participants who met the inclusion criteria and agreed to take part in this study. Due to the underlying disease (asthma), four participants were excluded from the study. As a result, 101 participants enrolled in this trial. After selection, the participants received an explanation of the objectives and study protocols. All participants volunteered to participate and provided written informed consent before data collection began.

### 2.2. Ethical Approval

The study was reviewed and approved by the University of Phayao Human Ethics Committee (protocol code UP-HEC 1.2/036/65 and date of approval 1 July 2022) before data collection began.

### 2.3. Data Collection

The participants were interviewed regarding demographic data, which included age, underlying disease, alcohol use, smoking, and exercise frequency. All participants were asked to complete the Suan Prung Stress Test-60 (SPST-60), a questionnaire for measuring stress levels. The physical fitness tests were applied to the participants once they finished the interview and stress assessment sections.

SPST-60 was used to measure the stress levels of the participants. SPST-60 is an instrument for which Cronbach’s alpha reliability is higher than 0.7, and its concurrent validity is higher than 0.27 (statistically significant based on electromyography values at a 95% CI) for the measurement of stress in the Thai population [21]. This questionnaire consists of three aspects and 60 items of stress assessment of the past six months. The first aspect (12 items) was designed to measure sensitivity or susceptibility to stress. The second aspect (24 items) was created to examine the sources of stress, such as work or study, personal, family, social, environmental, and financial. Lastly, the third aspect (24 items) was used to measure the symptoms of stress as manifested through skeletal muscles, parasympathetic and sympathetic nervous systems, emotion, cognition, and the endocrine and immune systems. The scores of each item were separated into five categories based on the frequency of the event or symptom. The participants were given only one category in each item for the frequency of events or symptoms that occurred to them in the past six months before participating in this study. In the susceptibility to stress aspect, the participants were scored 1 for an answer which always occurs, 2 for often, 3 for sometimes, 4 for rarely, and 5 for never (scoring rates were reversed for items 4—smoking, 5—alcohol consumption, and 6—taking sleeping pills or antidepressants). Conversely, for the sources of stress and the symptoms of stress aspects, the participants scored 5 for the answer of very high stress, 4 for high, 3 for average, 2 for low, and 1 for no stress. A higher total score means higher stress.

Anthropometric parameters, including the body mass index (BMI), waist-to-hip circumference ratio (WHR), and body fat percentage, were measured. Each participant’s weight and height were measured in order to calculate their BMI. BMI was calculated by dividing weight in kilograms by the square of height in meters [22]. WHRs (in centimeters) were assessed to calculate the WHR ratio. The waist circumference was measured midway between the costal margin and the iliac crest at the end of inspiration. Hip circumference was measured as the largest value above the buttocks. The waist circumference was then divided by the hip circumference to get the WHR [23]. The body fat percentage was measured indirectly using the skinfold thickness method. The skinfold thickness was measured by a caliper (Lange skinfold caliper, Beta Technology, Santa Cruz, CA, USA) at four sites, including the triceps, biceps, subscapular, and suprailiac crest. These measurements were then used to evaluate body fat by applying the equations estimated by Durnin and Womersley [24].

Cardiorespiratory fitness was assessed using the Queens College Step Test. For 3 min, the participants stepped on a 16 1/4-inch-high bench at a rate of 22 steps per minute. After the test, each participant remained standing. After 5 s of rest, the heart rate was measured for 15 s. The 15-s count was multiplied by 4 to get the beats per minute (bpm). The maximal oxygen consumption (milliliters/kilogram/minute; mL/kg/min) can be calculated [25].

Each participant’s muscular strength and flexibility were assessed for the musculoskeletal fitness test. The static strength of the grip squeezing muscles was measured using a grip strength dynamometer (T.K.K 5001 Grip-A, Takei Scientific Instruments, Niigata, Japan). Participants began by holding the hand grip with the dominant hand, standing erect, and keeping the arm straight and slightly abducted. The dynamometer was then squeezed as hard as possible with one brief maximal contraction and no extraneous body movement for 3 s. Grip strength was measured three times with a 1 min rest interval in between. The best score (kilograms) was divided by the participant’s body weight (kilograms) to determine relative strength [26]. Furthermore, we assessed the static strength of the leg muscles (Back and leg strength dynamometer, T.K.K 5402 Back-D, Takei Scientific Instruments, Niigata, Japan). The participants stood on the platform trunk erect, knees flexed 130–140 degrees and pronated, gripping the hand bar. The chain length was adjusted for each participant across the tight for positioning. The participants then slowly lifted the bar as far as they could while extending their knees. Two trials with a 1 min rest interval were performed. The best score of the leg muscle strength test was divided by the participant’s body weight to determine relative strength.

The modified sit-and-reach test was applied to measure participants’ flexibility (Standing trunk flexion meter, T.K.K. 5403 Flexion-D, Takei Scientific Instruments, Niigata, Japan). This test employs a 12-inch sit-and-reach box with a yardstick placed on top. Each participant sat on the floor with their buttocks, shoulders, and head against the wall, extended their knees, and pressed their feet against the box. The participant then reached forward slowly, sliding her fingers along the top of the yardstick. The fingertips’ most distant point on the yardstick was measured in centimeters [27].

Two physical therapy students who had received administration training from an experienced physical therapist administered the tests. The first evaluator was assessed using BMI, WHR, and the step test. Grip and leg muscle strength and muscle flexibility were tested by the second assessor.

### 2.4. Statistical Analysis

Descriptive statistics were used to present demographic data, stress levels, and physical fitness test results among female college students. All data were tested for a normally distributed pattern by the Kolmogorov–Smirnov test. The associations between stress levels and physical fitness levels were investigated using multiple linear regression analysis. Before being included in the regression model, all predictor variables were tested for significant covariates (*p* < 0.2). SPSS Statistics version 22.0 was used for all analyses. Statistical significance was defined as a *p*-value of 0.05.

## 3. Results

### 3.1. Demographic Characteristics of Female College Students

Table 1 displays demographic information. The subjects’ average age was 20.03 years (±1.36). All participants (100%) reported they did not smoke, and almost all (86.1%) reported they did not drink alcohol. About half (54.5%) of participants reported they exercised on a regular basis.

### 3.2. Stress Levels among Female College Students

SPST-60 was used to assess the participants’ stress levels. Table 2 shows the results. The mean susceptibility to stress score was in the moderate stress range (score between 21–26). The participants experienced high levels of stress from a variety of sources, including family, environment, and finances (scores between 8–13, 8–13, and 9–12, respectively). The mean score of total sources of stress was stated to be high (score between 58–79). Stress symptoms were prevalent in the parasympathetic and sympathetic nervous systems, as well as the endocrine and immune systems (scores between 5–9, 5–9, 5–9, and 5–10, respectively). Furthermore, the total stress symptom was stated to be high stress level (score between 37–57).

### 3.3. Physical Fitness Tests

Table 3 shows the results of the participants’ physical fitness tests. The mean body mass index was 21.73 kg/m^2^ (±4.56). The mean WHR was 0.79 (±0.09). The mean body fat percentage was 29.91% (±4.29). The mean maximal oxygen consumption was 41.47 mL/kg/min (±5.13). The participants’ mean relative hand grip strength was 0.46 (±0.08). The average relative leg strength was 1.16 (±0.38). Finally, the mean forward back flexibility was 19.79 cm (±11.53).

### 3.4. Association between Stress Levels Using SPST-60 Scores and Physical Fitness of Female College Students

Multiple linear regression analysis of the relationship between SPST-60 scores and physical fitness was performed, with potential covariates adjusted. Age and frequency of exercise were potential covariates. The results showed that sources of stress scores, here environment, were negatively associated with maximal oxygen consumption (β = −0.291; 95% CI, −0.551, −0.031). The results showed that symptoms of stress in the parasympathetic and sympathetic nervous systems were related to WHR (β = 0.010; 95% CI, 0.002, 0.017 and β = 0.006; 95% CI, 0.000, 0.012, respectively). Furthermore, the results showed that symptoms of stress, here emotion, were positively associated with the WHR (β = 0.005; 95%CI, 0.001, 0.009) and negatively associated with upper extremity muscle strength (β = −0.005; 95%CI, −0.009, 0.000) (Table 4).

## 4. Discussion

In this study, we assessed the stress levels of 101 female university students in the post-COVID-19 era. We also investigated the associations between stress levels as measured by the SPST-60 and physical fitness, including body composition, cardiorespiratory fitness, and musculoskeletal fitness. According to our survey, only 54.5% of participants exercised regularly during the experimental period, which was set in the first trimester of their return to onsite classes at the university. Sedentary lifestyles or low physical activity (PA) are linked to high stress levels [10,11,28]. Regular moderate-to-vigorous-intensity physical activity or exercise is proven to enhance both mental and physical health [29,30]. Reduced stress levels were associated with increased weekly PA frequency, particularly for women [10,11]. The present results showed that the participants had high stress levels in areas such as family, environment, and finances. The factors that might have induced the stress of the university student in the post-COVID-19 pandemic included the change in learning patterns from online to onsite classes, fear of becoming infected because the pandemic is not over, public transportation, a decline in family relationships, and economic disruption. However, stress levels are affected by a number of factors, such as the amount of stress, the duration of its influence, genetic components, and past illness history [3,31].

SPST-60 scores were also found to be significantly correlated with negative effects on maximum oxygen consumption, WHR, and hand grip strength. We used a 3 min step test to predict each participant’s maximum oxygen consumption for cardiorespiratory fitness. The results showed that stress scores in terms of sources of stress, here environment, were negatively related to maximal oxygen consumption (β = −0.291; 95% CI, −0.551, −0.031, *p* < 0.05). Our findings supported a previous study that found a significant negative relationship between cardiorespiratory fitness and stress levels in 824 normal-weight men [32]. According to Lee and Yang (2004) [33], psychological and physical stress have significant relationships with cardiopulmonary endurance in 139 adult women (*p* < 0.05 and *p* < 0.01, respectively). Another study reported that stress activates the autonomic nervous system, which in turn affects the cardiovascular system’s function [13]. The possible mechanism between stress and cardiorespiratory fitness could be explained by psychological stress stimulation via the alpha-adrenergic system, increasing heart rate and oxygen demand. Chronic stress causes vasoconstriction, which causes blood pressure to rise. High blood pressure causes the heart to work harder, resulting in left ventricle hypertrophy. These factors can contribute to a lack of cardiorespiratory fitness. Furthermore, an increase in blood pressure can result in cardiac arrhythmias and a subsequent myocardial infarction [12,13,14].

Our findings revealed the significant positive relationships between stress scores in terms of the symptoms of stress in the parasympathetic and sympathetic nervous systems and WHR (β = 0.010; 95% CI, 0.002, 0.017, *p* < 0.05, and β = 0.006; 95% CI, 0.000, 0.012, *p* < 0.05, respectively). Furthermore, the results showed that symptoms of stress, here emotion, had a positive association with WHR (β = 0.010; 95% CI, 0.002, 0.017, *p* < 0.05). In this study, we looked at the WHR as an indicator of fat mass or abdominal obesity. The WHR is a mathematical formula that can be used to identify obesity, abnormal fat distribution patterns, and the extent of abdominal adiposity. According to the birth cohort study, women who experienced one or more stressful life events had significantly higher central adiposity in both WHR and waist circumference [34]. Kubera et al. (2017) investigated statistical trends that linked women with high psychological distress to changes in body form and an increased WHR [19]. In addition, Hyatt et al. (2019) discovered a negative relationship between percent body fat and perceived stress in female volleyball players (r = 0.34, *p* < 0.05) [20]. The following list outlines a potential mechanism that could account for the correlations between stress and WHR. Chronic stress has been linked to both psychological and physical symptoms, most notably increased fat mass and chronic systemic inflammation. These cause adipocyte hypertrophy and hyperplasia, alter adipokine secretion, and attract and activate stromal fat immune cells [35,36,37]. Stress activates the hypothalamic-pituitary-adrenal (HPA) axis and sympathetic nervous system, resulting in hormonal abnormalities such as increased cortisol secretion and visceral fat development [38]. Cortisol is a hormone that acts as a neurotransmitter in the brain. Cortisol oscillates in a 24 h cycle. While the blood cortisol level is highest in the early morning, it reaches its lowest point at midnight. The bodily function can be affected by prolonged activation of the stress response system and excessive cortisol exposure. This raises the risk of numerous health issues, such as increased muscle tension and pain, heart disease, sleep problems, memory loss, and weight gain [39]. Changes in eating behavior are also caused by hyperactivation of the HPA axis [40,41]. When people are exposed to chronic stressors, eating control is lost as a result of the hedonic reward of eating as a counter-maladaptation to stress dysphoria [42]. These factors have an impact on dietary intake, such as the consumption of energy-dense foods, which may increase fat mass and WHR [18].

Our results also showed a significant negative relationship between symptoms of stress, here emotion, and hand grip strength as measured by the hand grip dynamometer (β = −0.005; 95% CI, −0.009, 0.000, *p* < 0.05). However, we did not find a significant correlation between stress levels and leg strength. This might be caused from that the upper limbs are often used for fine motor skills, such as writing, typing, or manipulating small objects, while the lower limbs are used for gross motor skills, such as running, jumping, or lifting heavy objects. Young adults tend to use their lower limb muscles more than their upper limb muscles to perform daily activities. For example, walking to work or the classroom requires the use of the lower limbs to cover the distance. As a result, university students might have a decline in upper limb muscle strength rather than lower limb. Our findings were consistent with previous research, which found a statistically significant negative relationship between stress and muscle strength as measured by maximum voluntary contraction of dominant hand grip strength (r = −0.0675, *p* = 0.000) [43]. In addition, Wang et al. (2022) found a negative correlation between grip strength and depression (OR = 1.237; 95% CI, 1.172–1.305) [44]. The authors suggested that hypotonia may be related to the abrupt development of depressive symptoms. In addition, stress hormones lead to muscular protein catabolism, which reduces muscle strength. The following factors can be used to comprehend the precise mechanism. The detrimental effects of chronic stress, hypercortisolism, and low-grade systemic inflammation on muscle mass lead to mitochondrial dysfunction. These variables all lead to a loss in skeletal muscle quality, strength, and function [45,46,47]. Nevertheless, any restrictions on mental capacity, performance level, or muscle strength are not only the result of stress but also insufficient physical activity. 

Our study had some limitations. First, a cause-and-effect link cannot be investigated in this cross-sectional study, nor can the direction of the associations be determined. Second, the factors associated with stress levels were not identified, such as onsite class duration, financial distress, and fear of infection, with the exception of demographic characteristics. Third, the responses to the stress of each participant differed depending on their experience and beliefs. Finally, we only collected data from females. All limitations should be considered to improve a future study.

## 5. Conclusions

According to the findings of this study, high stress levels among female university students post-COVID-19 pandemic were associated with an increase in WHR. Furthermore, stress levels were found to be inversely related to maximal oxygen consumption and upper extremity muscle strength. As a result, stress reduction or prevention alternatives should be considered in order to maintain physical fitness and prevent stress disorders. In addition, female university students should be encouraged to exercise regularly, incorporating both aerobic and strengthening exercises.

## Figures and Tables

**Table 1:** Demographic characteristics of female college students (*n* = 101).

Parameters		*n* (%) or Mean ± SD
Age (years), mean ± SD		20.03 ± 1.36
Underlying disease, *n* (%)		0 (0)
Smoking, *n* (%)		0 (0)
Alcohol use, *n* (%)		14 (13.9)
Exercise frequency, *n* (%)	<3 days/week or no exercise	46 (45.5)
	≥3 days/week	55 (54.5)

Note: SD standard deviation.

**Table 2:** Suan Prung Stress Test-60 (SPST-60) scores of female college students (*n* = 101).

SPST-60 Aspects	Mean	Median	SD	Range
Susceptibility to stress	25.26	25	5.36	14–43
Sources of stress				
Work or study	19.69	20	5.14	9–33
Personal	9.49	9	2.99	4–18
Family	8.31	8	3.82	4–20
Social	4.92	5	1.64	2–9
Environment	8.60	8	3.36	4–17
Financial	8.54	8	3.17	3–15
Total sources of stress	59.55	58	16.24	26–107
Symptom of stress				
Skeletal muscles system	6.97	7	2.66	3–13
Parasympathetic nervous system	5.33	5	2.21	2–12
Sympathetic nervous system	5.85	5	2.85	3–15
Emotion	9.10	9	3.79	4–20
Cognition	7.01	7	2.85	3–15
Endocrine system	8.32	8	3.24	4–20
Immune system	6.52	6	2.50	4–13
Total symptoms of stress	49.70	47	17.29	24–106

Note: SPST-60 Suan Prung Stress Test-60; SD standard deviation.

**Table 3:** The results of physical fitness tests (*n* = 101).

Physical Fitness Tests	Mean	Median	SD	Range
Body mass index (kg/m^2^)	21.73	20.61	4.56	15.06–41.85
Waist-to-hip circumference ratio	0.79	0.78	0.09	0.68–1.43
Body fat (%)	29.91	30.30	4.29	21.40–38.00
Maximal oxygen consumption (mL/kg/min)	41.47	42.17	5.13	31.27–52.70
Relative hand grip strength	0.46	0.47	0.08	0.25–0.63
Relative leg strength	1.16	1.11	0.38	0.57–2.57
Forward back flexibility (cm)	19.79	16.26	11.53	5.08–50.80

Note: SD standard deviation; kg/m^2^ kg per square meter; mL/kg/min milliliters per kilogram per minute; cm centimeter.

**Table 4:** Association between stress levels using SPST-60 scores and physical fitness of female university students (*n* = 101).

SPST-60 Aspect	β (95% CI)						
	BMI	WHR	Body Fat	VO_2_max	Hand Grip Strength	Leg Strength	Flexibility
Susceptibility to stress	−0.026 (−0.211, 0.159)	−0.002 (−0.005, 0.002)	−0.040 (−0.214, 0.133)	0.007 (−0.025, 0.038)	0.001 (−0.003, 0.004)	0.009 (−0.006, 0.024)	−0.163 (−0.474, 0.148)
Sources of stress							
Work or study	0.060 (−0.119, 0.239)	0.002 (−0.001, 0.006)	0.135 (−0.031, 0.302)	−0.041 (−0.215, 0.133)	−0.003 (−0.006, 0.000)	−0.007 (−0.021, 0.008)	0.069 (−0.372, 0.234)
Personal	0.068 (−0.245, 0.381)	0.003 (−0.003, 0.009)	0.084 (−0.210, 0.378)	−0.082 (−0.386, 0.222)	−0.003 (−0.009, 0.003)	−0.016 (−0.042, 0.009)	−0.345 (−0.870, 0.180)
Family	−0.092 (−0.332, 0.149)	−0.001 (−0.006, 0.003)	−0.066 (−0.292, 0.160)	0.002 (−0.232, 0.237)	−0.002 (−0.006, 0.003)	−0.002 (−0.022, 0.018)	−0.129 (−0.536, 0.278)
Social	0.167 (−0.396, 0.730)	0.009 (−0.001, 0.020)	0.171 (−0.358, 0.700)	−0.308 (−0.852, 0.236)	−0.007 (−0.017, 0.003)	−0.035 (−0.081, 0.011)	0.007 (−0.946, 0.960)
Environment	−0.019 (−0.294, 0.255)	−0.001 (−0.006, 0.004)	0.064 (−0.194, 0.322)	−0.291 (−0.551, −0.031) *	−0.686 (−0.005, 0.005)	−0.007 (−0.029, 0.016)	0.161 (−0.303, 0.624)
Financial	0.138 (−0.154, 0.430)	−0.005 (−0.010, 0.001)	0.007 (−0.269, 0.282)	−0.116 (−0.399, 0.168)	−0.004 (−0.009, 0.001)	−0.004 (−0.028, 0.020)	0.003 (−0.493, 0.498)
Total sources of stress scores	0.009 (−0.048, 0.066)	0.000 (−0.001, 0.001)	0.018 (−0.036, 0.071)	−0.027 (−0.082, 0.028)	−0.001 (−0.002, 0.000)	−0.002 (−0.007, 0.003)	−0.019 (−0.115, 0.078)
Symptoms of stress							
Skeletal muscles system	−0.030 (−0.383, 0.324)	0.000 (−0.007, 0.006)	0.096 (−0.235, 0.428)	−0.141 (−0.483, 0.201)	−0.003 (−0.010, 0.003)	−0.026 (−0.054, 0.003)	−0.234 (−0.830, 0.361)
Parasympathetic nervous system	0.119 (−0.299, 0.536)	0.010 (0.002, 0.017) *	0.183 (−0.208, 0.574)	−0.200 (−0.604, 0.204)	−0.003 (−0.011, 0.004)	−0.005 (−0.039, 0.029)	−0.419 (−1.120, 0.282)
Sympathetic nervous system	0.036 (−0.286, 0.357)	0.006 (0.000, 0.012) *	0.152 (−0.149, 0.453)	−0.097 (−0.409, 0.214)	−0.003 (−0.008, 0.003)	−0.015 (−0.041, 0.011)	−0.293 (−0.833, 0.247)
Emotion	0.140 (−0.102, 0.381)	0.005 (0.001, 0.009) *	0.138 (−0.089, 0.364)	−0.012 (−0.247, 0.224)	−0.005 (−0.009, 0.000) *	−0.012 (−0.032, 0.007)	−0.217 (−0.625, 0.191)
Cognition	0.180 (−0.148, 0.508)	0.005 (−0.001, 0.011)	0.212 (−0.095, 0.520)	0.028 (−0.292, 0.349)	−0.005 (−0.011, 0.001)	−0.017 (−0.044, 0.010)	−0.035 (−0.593, 0.522)
Endocrine system	−0.026 (−0.312, 0.261)	−0.001 (−0.006, 0.005)	0.027 (−0.242, 0.295)	−0.112 (−0.389, 0.165)	0.000 (−0.005, 0.006)	−0.005 (−0.029, 0.018)	0.002 (−0.482, 0.486)
Immune system	0.003 (−0.365, 0.371)	0.002 (−0.005, 0.009)	−0.060 (−0.406, 0.285)	−0.150 (−0.506, 0.206)	−0.002 (−0.008, 0.005)	−0.027 (−0.057, 0.003)	0.229 (−0.391, 0.849)
Total symptoms of stress scores	0.006 (−0.048, 0.059)	0.001 (0.000, 0.002)	0.021 (−0.029, 0.071)	−0.017 (−0.069, 0.034)	0.000 (−0.001, 0.000)	−0.003 (−0.007, 0.002)	−0.037 (−0.127, 0.053)

Note: β beta; 95% CI 95% confidence interval; SPST-60 Suan Prung Stress Test-60; BMI body mass index; WHR waist-to-hip circumference ratio; VO_2_ max maximal oxygen consumption; * *p* < 0.05.

## Data Availability

Data is unavailable due to ethical restrictions.
